# Hey2 functions in parallel with Hes1 and Hes5 for mammalian auditory sensory organ development

**DOI:** 10.1186/1471-213X-8-20

**Published:** 2008-02-26

**Authors:** Shuangding Li, Sharayne Mark, Kristen Radde-Gallwitz, Rebecca Schlisner, Michael T Chin, Ping Chen

**Affiliations:** 1 Department of Cell Biology, Emory University School of Medicine, Atlanta, GA 30322, USA; 2 University of Washington School of Medicine at SLU, 815 Mercer Street, Seattle, WA 98109, USA

## Abstract

**Background:**

During mouse development, the precursor cells that give rise to the auditory sensory organ, the organ of Corti, are specified prior to embryonic day 14.5 (E14.5). Subsequently, the sensory domain is patterned precisely into one row of inner and three rows of outer sensory hair cells interdigitated with supporting cells. Both the restriction of the sensory domain and the patterning of the sensory mosaic of the organ of Corti involve Notch-mediated lateral inhibition and cellular rearrangement characteristic of convergent extension. This study explores the expression and function of a putative Notch target gene.

**Results:**

We report that a putative Notch target gene, hairy-related basic helix-loop-helix (bHLH) transcriptional factor Hey2, is expressed in the cochlear epithelium prior to terminal differentiation. Its expression is subsequently restricted to supporting cells, overlapping with the expression domains of two known Notch target genes, *Hairy *and enhancer of split homolog genes *Hes1 *and *Hes5*. In combination with the loss of *Hes1 *or *Hes5*, genetic inactivation of *Hey2 *leads to increased numbers of mis-patterned inner or outer hair cells, respectively. Surprisingly, the ectopic hair cells in *Hey2 *mutants are accompanied by ectopic supporting cells. Furthermore, *Hey2*^-/-^*;Hes1*^-/- ^and *Hey2*^-/-^*;Hes1*^+/- ^mutants show a complete penetrance of early embryonic lethality.

**Conclusion:**

Our results indicate that *Hey2 *functions in parallel with *Hes1 *and *Hes5 *in patterning the organ of Corti, and interacts genetically with *Hes1 *for early embryonic development and survival. Our data implicates expansion of the progenitor pool and/or the boundaries of the developing sensory organ to account for patterning defects observed in *Hey2 *mutants.

## Background

The organ of Corti consists of four parallel rows of sensory hair cells along the longitudinal axis of the spiraled cochlea. The first row of hair cells from the center, or the medial side, of the cochlea are known as inner hair cells (IHCs). The remaining three rows of hair cells toward the periphery, or the lateral side, of the cochlea are known as outer hair cells (OHCs). Invariably, hair cells are separated from each other by several types of morphologically distinct non-sensory supporting cells. The mosaic cellular arrangement in the organ of Corti allows the tissue to sustain mechanic transduction and to maintain homeostasis required for the function and survival of the organ of Corti.

The precise cellular architect of the organ of Corti provides a unique system to examine mechanisms underlying cellular patterning. During development, the precursor cells that give rise to both the sensory hair cells and supporting cells of the organ of Corti exit the cell cycle around embryonic day 14.5 (E14.5) in mice [1,2]. Subsequently, hair cell differentiation initiates near the base of the cochlea at the inner hair cell location and gradually reaches the ends of the cochlear duct along the longitudinal axis and toward the outer-most row of outer hair cells laterally [2-4]. The differentiation of hair cells requires a basic helix-loop-helix (bHLH) protein, atonal homolog 1 (Atoh1) or mouse atonal homolog 1 (Math1) [5]. The differentiation of the supporting cells appears to follow that of hair cells [6]. The molecular mechanism underlying the mosaic arrangement of hair cells and supporting cells has been shown to involve Notch signaling-mediated lateral inhibition [7-9]. During Notch signaling, the expression of a transcription activating bHLH gene, such as *Math1*, leads to the expression of Notch ligands at hair cell membranes [10,11]. Notch ligands bind to the receptor Notch on neighboring cells to activate Notch [10,11]. As a consequence of Notch activation, hairy and enhancer of split (Hes) homologs or hairy-related (Hey) bHLH transcriptional repressors [12] are expressed, which suppress the expression of the activating bHLH gene in these neighboring cells [10,11,13,10,11,13-15]. The neighboring cells therefore adopt a different cell fate. In zebrafish, mutations in Notch signaling transform inner ear sensory organs into epithelia consisting entirely of hair cells [16], supporting Notch-mediated lateral inhibition in patterning sensory mosaics of the inner ear. In mice, components of Notch signaling, such as Notch ligands Delta-like1 (Dll1) and Jagged2 (Jag2), Notch1, Hes1 and Hes5, are expressed in the organ of Corti during terminal differentiation of the organ of Corti [14,15,17-20]. Consistent with their expression in the cochlear epithelium, loss-of-function of these genes leads to mis-patterning of the hair cells and variously increased numbers of hair cells [7,8,14,15,19]. These data together indicates that Notch signaling regulates cellular patterning of inner ear sensory organs in mammals.

Patterning defects within Notch mutants vary greatly. In particular, *Notch1 *mutants and *Dll1*; *Jag2 *double mutants have greatly increased numbers of hair cells and drastically altered patterning of the organ of Corti [7,8] while *Hes1 *and *Hes5 *mutants have much weaker patterning defects [14,15], implicating compensatory pathways for *Hes1 *and *Hes5 *in the organ of Corti. To explore compensating genes for Hes1 and Hes5, we used differential gene expression profiling and candidate gene approaches and identified the expression of two additional Hairy-related genes, *Hey1 *and *Hey2 *[21-23], in the cochlea during terminal differentiation of the organ of Corti. We further analyzed the function of Hey2 in the development of the organ of Corti.

## Results

### Dynamic expression of *Hey2 *in the developing inner ear

**Figure 1:**
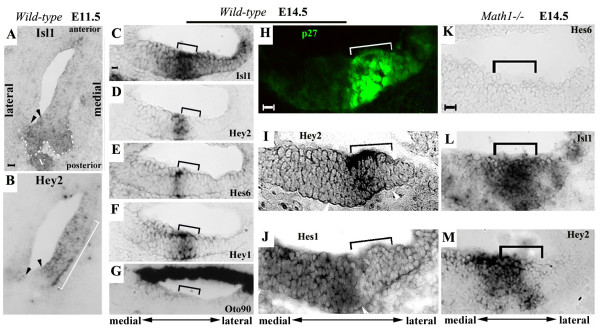
**Expression of *Hey2 *in the developing inner ear**. Otocyst (**A**-**B**) or cochlear sections (**C**-**J**) from E11.5 (**A**-**B**), E14.5 (**C**-**J**) were hybridized with RNA probes for *Isl1 *(**A**,**C**), *Hey2 *(**B**,**D**,**I**), *Hes1 *(**J**), *Hes6 *(**E**), *Hey1 *(**F**), and *Oto90 *(**G**). Arrowheads in **A**-**B **indicate the domain for sensory and neuronal lineages in the otocyst; the bracket in **B **marks the expression domain of *Hey2*; brackets in **C**-**J **indicate the primordial organ of Corti at E14.5. (**K**-**M**) Cochlear sections from an E14.5 *Math1*^-/- ^embryo were probed for Hes6 (**K**), Isl1 (**L**), and Hey2 (**M**). Brackets indicate the developing sensory organ that was determined by careful comparison of adjacent sections with molecular markers for the region. The sides of the cochlear duct toward the center and the periphery of the spiraled cochlea are designated as the medial and lateral sides, respectively, and are indicated. Scale bars: 10 μm for **A**-**B**, **C**-**G**, **H**-**J**, **K**-**M**.

The inner ear is derived from the otic placode near the hindbrain. In mice, the otic placode invaginates to form an enclosed otocyst by E10.5. By E11.5, specific molecular markers are expressed in regions that are designated for different cell lineages [24]. In particular, transcriptional factor Islet1 (Isl1) marks the sensory and neuronal lineages of the inner ear at early otocyst stages (Fig. 1A) [24]. Message RNA for Otoconin90 (Oto90) [25], a major protein component of murine otoconia (composite crystals that overlie the gravity sensory receptors in the vestibular macular organs), is localized to anterior and lateral regions of the otocyst (data not shown). We compared the expression of *Hey2 *at E11.5 to that of *Isl1 *and *Oto90 *(Fig. 1A, B, and data not shown). *Hey2 *is expressed in the medial region of the otic epithelium (Fig. 1B). Its expression abuts *Isl1*-expressing and *Oto90*-expressing domains at its posterior and anterior boundaries, respectively (Fig. 1A, B, and data not shown). Therefore, at early otocyst stages, the expression of *Hey2 *appears to be complementary to the sensory and neuronal lineages of the inner ear and excluded from cells expressing *Oto90*.

**Figure 2:**
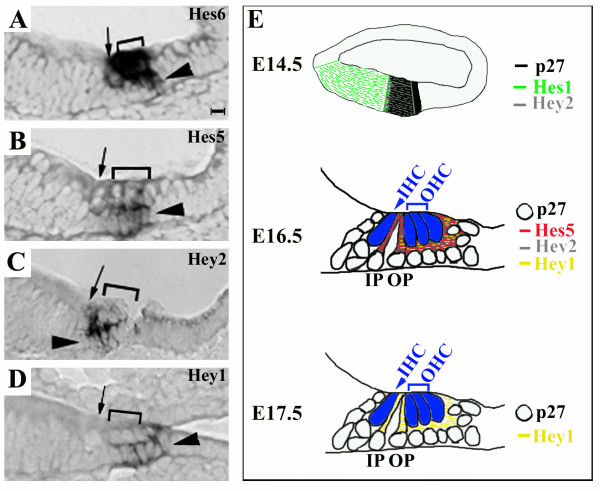
**Hey2 is expressed in supporting cells in the developing organ of Corti**. Adjacent cochlear sections (**A**-**D**) from an E16.5 embryo were hybridized with probes against *Hes6 *(**A**), *Hes5 *(**B**), *Hey2 *(**C**) or *Hey1 *(**D**). The inner (arrows in **A**-**D**) and outer hair cell regions (brackets in **A**-**D**) are indicated. The arrows in **A**-**D **indicate the layer of supporting cells. (**E**) The dynamic expression of *Hey2 *in the developing cochlea is schematically diagramed. At E14.5, the expression domain of *Hey2 *(grey lines) overlaps with that of *Hes1 *(green lines) and p27/Kip1 (black) at its medial and lateral borders, respectively. At E16.5, *Hey2 *is expressed similarly to that of *Hes5 *(red lines) and *Hey1 *(yellow lines) in a subset of supporting cells, while p27/Kip1 is present in all the cells surrounding hair cells (open circles). By E17.5, the expression of *Hey2 *has started to be down-regulated from the organ of Corti at the basal region where the expression of *Hey1 *remains. Scale bar: 10 μm for **A**-**D**.

We further examined the expression of *Hey2 *in later stages in the cochlea (Figs. 1 and 2). At E14.5, the precursor cells that will give rise to hair cells and supporting cells of the organ of Corti are post-mitotic and are demarcated by the expression of a cyclin-dependent kinase inhibitor, p27/Kip1 (Figs. 1H, 2E) [2]. The expression of *Isl1 *also marks the primordial organ of Corti (Figs. 1C, 2E) [24]. At this stage, a Notch target gene *Hes1 *is expressed in a broad region medial to the sensory region (Figs. 1J, 2E) [14,15], while the expression of *Hes6 *has begun at the medial boundary of the sensory primordium and marks the differentiating inner hair cells (Fig. 1E) [26]. The expression of *Oto90 *is restricted to the roof of the cochlear duct (Fig. 1G). The expression of *Hey2 *appears in a region overlapping with the developing organ of Corti (Figs. 1D, 1I, 2E). Careful comparisons with different molecular markers in the adjacent sections led us to conclude that the expression domain of *Hey2 *may be shifted slightly to the medial side of the developing organ of Corti marked by the expression of *Isl1 *and p27/Kip1 (compare Fig. 1C with 1D, and compare Fig. 1H with 1I). In addition, another Hey gene [23], *Hey1*, is expressed in the primordial organ of Corti at this stage (Fig. 1F). In contrast to *Hey2 *and *Hey1 *and *Hes1*, *Hes5 *was not detected in the cochlea at E14.5 (data not shown).

Following the establishment of the post-mitotic primordial organ of Corti, the differentiation of hair cells and supporting cells is initiated near the base of the cochlea at the inner hair cell region [2,27]. By E16.5, the organ of Corti in the basal to medial regions of the cochlea has been patterned into a sensory mosaic consisting of one row of inner and three rows of outer hair cells interdigitated with supporting cells (Fig. 2). From a cross section of the cochlear duct, hair cells are seen at the lumenal layer while the nuclei of supporting cells are localized to the basal layer of the cochlear epithelium (Fig. 2A–D and the diagrams in 2E), and the cytoplasmic phalangeal processes of supporting cells project into the lumenal hair cell layer to separate hair cells from each other. We compared the expression of a basic helix-loop-helix gene, Hes6, in the cochlea at E16.5 to several other molecular markers. As we have previously shown [26], *Hes6 *is restricted to the hair cells at the lumenal layer at this stage (Fig. 2A). In contrast, *Hes5 *message RNA is limited to the supporting cell nuclei (Fig. 2B) [14]. Similarly, the expression of *Hey2 *(Fig. 2C) and *Hey1 *(Fig. 2D) is also seen in the supporting cells. By E17.5, the expression of Hey2 has been down-regulated starting from the base of the cochlea to the apex, while the expression of *Hey1 *still remains in the supporting cells (data not shown, diagramed in Fig. 2E).

The early onset of *Hey2 *in the developing inner ear at E11.5 and the broad expression of *Hey2 *in the cochlea at E14.5 suggest that its initial expression might precede hair cell and supporting cell differentiation in the cochlea. To test this possibility, we examined the expression of *Hey2 *in *Math1*^-/- ^animals [5], and compared its expression with that of *Hes6 *and *Isl1 *(Fig. 1D, F). In the absence of Math1, the primordial organ of Corti marked by the expressed of p27/Kip1 is established [27]. However, hair cells fail to differentiate and cells within the primordial organ of Corti die [27]. Hes6 marks the sensory lineage in the developing inner ear and its expression in cochlear hair cells depends on Math1 [26]. In *Math1*^-/- ^cochleae, *Hes6 *was not expressed (Fig. 1K). *Isl1 *is expressed early in the otocyst and its expression in the organ of Corti is independent of Math1 (Fig. 1L) [24]. Similar to *Isl1*, *Hey2 *was expressed in the otocyst and its expression was maintained in the cochlear epithelium in *Math1*^-/- ^animals at E14.5 (Fig. 1M).

In summary, *Hey 2 *is expressed in the otocyst prior to the formation of the cochlear duct; its onset in the cochlea is independent of Math1; and its expression in the cochlea is dynamic, partially overlapping with *Hes1 *and *Hes5 *in the developing cochlea (Fig. 2E). An additional Hey gene, *Hey1*, is also expressed in an overlapping pattern with *Hey2*.

### Hey2 plays a role in patterning outer hair cells

**Figure 3:**
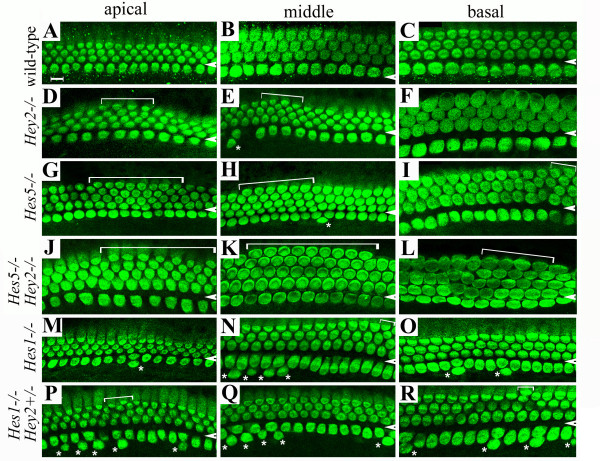
**Hey2 functions in parallel with Hes5 and Hes1 in regulating outer hair cell and inner hair cell patterning, respectively**. Cochlear whole mounts were prepared from wild-type (**A**-**C**), *Hey2*^-/- ^(**D**-**F**), *Hes5*^-/- ^(**G**-**I**), *Hes5*^-/- ^;*Hey2*^-/- ^(**J**-**L**), *Hes1*^-/- ^(**M**-**O**), and *Hes1*^-/-^*;Hey2*^+/- ^(**P**-**R**) embryos at E18.5, and stained for a hair cell marker, Myosin6. Images from the apical (**A**, **D**, **G**, **J, M, P**), middle (**B**,**E**,**H**,**K, N, Q**), and basal (**C**,**F**,**I**,**L, O, R**) regions of the cochlea along the longitudinal axis were included. Arrowheads indicate the region that separates the inner from outer hair cells. Extra rows of outer hair cells (**D**,**E**,**G**-**L, N, P, **indicated by brackets) and inner hair cell doublet (**E, H, M-R, **indicated by asterisks) were seen in mutant samples in comparison to the wild-type control (**A**-**C**). Scale bar: 10 μm.

**Figure 4:**
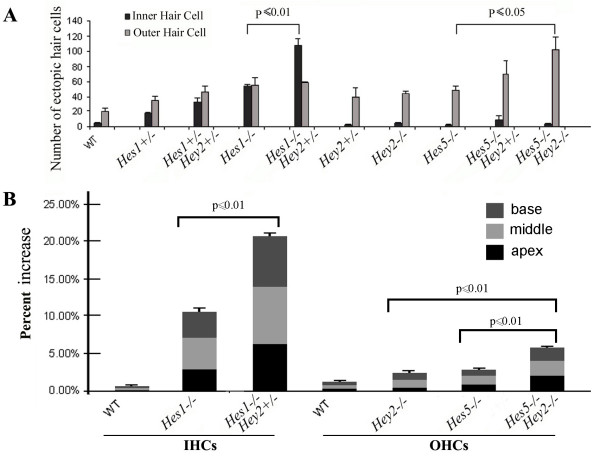
**Quantifications of hair cell patterning defects in *Hey2 *mutants**. Extra rows of outer hair cells and inner hair cell doublets were quantified in the cochleae isolated from animals with the genotypes specified (**A**). The percent of the number of hair cells in extra rows was calculated against the number of normally patterned hair cells in the same cochlea, and plotted for each genotype specified in the graph (**B**). The distribution of mis-patterned hair cells along the longitudinal axis of the cochlea was indicated (**B**). Note the uniform increase of ectopic hair cells along the longitudinal axis of the cochlea in *Hey2*^-/-^*;Hes5*^-/- ^and *Hey2*^+/-^*;Hes1*^-/- ^mutants. The p values for statistic significance among the groups in each bracket are indicated (**A**,**B**).

To examine whether the dynamic expression of *Hey2 *in the inner ear is required for inner ear development, we examined the loss-of-function of Hey2 in mice. *Hey2*^-/- ^animals die around postnatal day 5 (P5) due to massive postnatal cardiac hypertrophy and isolated ventricular septal defects [28-30]. We therefore examined inner ears from *Hey2*^-/- ^animals from E13.5 to P2. Morphologically, no visible abnormality was observed in inner ears isolated from *Hey2*^-/- ^animals at all the stages examined (data not shown). Upon molecular examinations using cell type-specific antibodies, we consistently observed a minor patterning defect in the organ of Corti from *Hey2*^-/- ^animals (Figs. 3 and 4). In comparison to the normal three rows of outer hair cells in wild-type littermates (Fig. 3A–C), cochlear whole mount preparations from *Hey2*^-/- ^animals stained for hair cell-specific marker Myosin6 showed segments of an additional row of outer hair cells (Figs. 3D–F, 4, mis-patterned OHCs = 43.6 ± 5.6, n = 3). The apparent patterning defect in outer hair cells in *Hey2*^-/- ^animals is reminiscent of that observed in *Hes5*^-/- ^animals [14,15], and the number of additional outer hair cells in *Hey2*^-/- ^animals is comparable to that observed in *Hes5*^-/- ^(Fig. 3G–I, mis-patterned OHCs = 47.6 ± 9.8, n = 3) animals among littermates (Figs. 3D–I, 4, p = 0.574).

*Hey2 *belongs to the same bHLH gene sub-family as *Hes5*, which can function as downstream targets for Notch signaling [31]. The similarity in patterning defect observed in *Hey2*^-/- ^and *Hes5*^-/- ^animals suggests that Hey2 may play a similar role as Hes5 in the organ of Corti. The partially overlapping expression of *Hey2 *and *Hes5 *(Fig. 2B, C) during terminal differentiation of the organ of Corti and the weak patterning defect in *Hey2*^-/- ^or *Hes5*^-/- ^animals further suggested that Hey2 may function redundantly with Hes5 for patterning the organ of Corti. To test this possibility, we crossed animals carrying mutant alleles for both *Hey2 *and *Hes5*, and compared *Hey2*^-/- ^(Fig. 3D–F), *Hes5*^-/- ^(Fig. 3G–I), and *Hey2*^-/-^*; Hes5*^-/- ^(Fig. 3J–L) littermates, and quantified the number of mis-patterned outer hair cells in these animals (Fig. 4). We found a statistically significant increase in the numbers of mis-patterned outer hair cells in *Hey2*^-/-^*; Hes5*^-/- ^animals (mis-patterned OHCs = 101.3 ± 29.1, n = 3) in comparison to *Hey2*^-/- ^(p = 0.028) or *Hes5*^-/- ^(p = 0.039) animals (Fig. 4). The effect of genetic inactivation of *Hey2 *and *Hes5 *on the patterning of outer hair cells appeared to be additive (Fig. 4).

### Hey2 functions in parallel with Hes1 in patterning inner hair cells

In addition to the presence of segments of additional row of outer hair cells, we also observed variable amounts of inner hair cell doublets in *Hey2*^-/- ^animals (Fig. 3D–F), in contrast to a single row of inner hair cells in control animals (Fig. 3A–C). However, statistic analyses showed that there is no significant increase in the number of inner hair cell doublets in *Hey2*^-/- ^animals (IHC doublets = 4 ± 3, n = 3) in comparison to wild-type (IHC doublets = 3 ± 2, n = 3) control littermates (Fig. 4, p = 0.768). Since *Hey2 *shows a partial overlapping expression with a homologous gene, *Hes1*, at the onset of terminal differentiation of the organ of Corti (Fig. 1I, J) and the loss of *Hes1 *results in an increase in inner hair cell doublets and, to a lesser degree, an additional row of outer hair cells (Fig. 3M–O) [14,15], we sought to determine whether Hey2 plays a redundant role to Hes1 in development of the organ of Corti.

As reported previously, *Hes1*^-/- ^animals show progressively increased lethality after E12.5 [31]. We found that *Hes1*^-/-^*; Hey2*^-/- ^animals have a complete penetrance in early embryonic lethality. All of the *Hes1*^-/-^*; Hey2*^-/- ^animals died before E13.5, prior to the terminal differentiation of the organ of Corti. In addition, in littermate crosses between *Hes1*^+/-^*; Hey2*^+/- ^double heterozygotes and *Hey2*^+/- ^single heterozygotes or within *Hes1*^+/-^*; Hey2*^+/-^double heterozygotes, eight *Hes1*^+/-^*; Hey2*^-/- ^embryos were expected, none of which were recovered after E13.5, indicating a strong genetic interaction between Hey2 and Hes1 for early development and survival of mouse embryos.

We were able to recover *Hes1*^-/- ^and *Hes1*^-/-^*; Hey2*^+/- ^embryos and analyzed organs of Corti isolated from these animals (Figs. 3, 4). In comparison to *Hes1*^-/- ^animals that showed frequent inner hair cell doublets (Figs. 3M–O, 4, IHC doublets = 56.7 ± 1.5, n = 3), the loss of a single copy of *Hey2 *in the *Hes1*^-/- ^background (Figs. 3P–R, 4, IHC doublets = 108.3 ± 15.2, n = 3) led to a 2 fold-increase in the number of inner hair cell doublets (Fig. 4, p = 0.0003). Since *Hey2*^+/- ^had no more inner hair cell doublets than control samples (Fig. 4, IHC doublets = 1.6 ± 1.4, n = 3), the increase in inner hair cell doublets in *Hes1*^-/-^*; Hey2*^+/- ^animals appeared to be synergistic rather than additive. These data together indicate that *Hey2 *genetically interacts with *Hes1 *for early development of mouse embryos and for patterning inner hair cells in the organ of Corti.

### Ectopic hair cells in *Hey2 *compound mutant animals are accompanied by an increase in surrounding supporting cells

The mis-patterning of hair cells in *Hes1 *and *Hes5 *mutants was observed previously [14,15]. It was thought that the conversion of supporting cells to hair cells might account for the abnormality observed in *Hes1 *and *Hes5 *mutants, as predicted from a simple lateral inhibition model [14,15]. However, the underlying molecular mechanism has not been tested. We observed enhanced phenotypes in *Hes1 *and *Hey2 *and in *Hes5 *and *Hey2 *compound mutants (Figs, 3 and 4), and explored the molecular roles of Hes family genes in patterning the organ of Corti.

**Figure 5:**
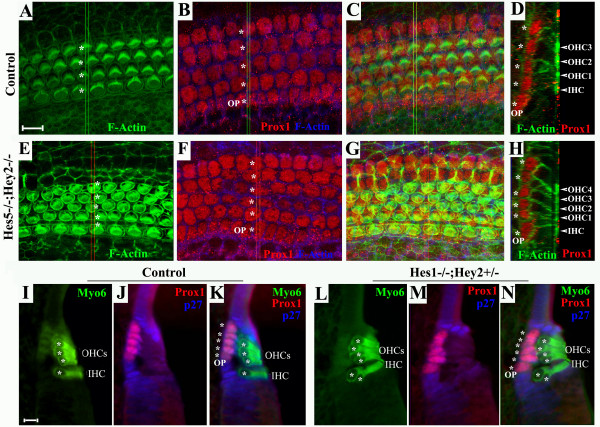
**Ectopic supporting cells associated with ectopic hair cells in *Hey2 *mutants**. Cochlear whole mount preparations from E18.5 wild-type control (**A**-**C**) and *Hes5*^-/-^*;Hey2*^-/- ^(**E**-**G**) animals were stained for F-actin (phalloidin, green) and Prox1 (red). Confocal Z-scans were taken to capture F-actin labeling from the apical surface (**A**,**E**) to Prox1 labeling at the layer of supporting cell nuclei (**B**,**F**). The Z-scans were stacked to project both labels in the organ of Corti (**C**,**G**). Orthogonal cross sections of the projections of the organ of Corti stained with F-actin and Prox1 were selected to view the relative positions of hair cells (**D**,**H**), in which stereocilia and cellular cortex were labeled strongly by F-actin, and the nuclei of supporting cells were labeled by Prox1. The positions where the orthogonal sections were taken are indicated by double green lines (**B**,**C**) or double red lines (**F**,**G**). Note that precise patterning 4 rows of hair cells and 5 rows of Prox1^+ ^supporting cells is seen in the control preparation (**A**-**D**), while ectopic hair cells in the *Hes5*^-/-^*;Hey2*^-/- ^preparation are associated with ectopic Prox1^+ ^supporting cells (**E**-**H**). (**I**-**N**) Cross sections from E18.5 control and *Hes1*^-/-^*;Hey2*^+/- ^embryos were stained for Myosin6 (**I**,**L, **Myo6), Prox1 (**J**,**M**), and p27/Kip1 (**J**, **M**). **K **and **N **are overlays of the triple stains for each sample. The asterisks mark hair cells (**A**,**I**,**L**), supporting cells (**B**,**D**,**F**,**H**), or hair cells and supporting cells (**K**,**N**). OP: outer pillar cell; OHC: outer hair cells; IHC: inner hair cell. Scale bars: 10 μm for **A**-**H**, **I**-**N**.

We examined the cellular architect of the organ of Corti in control and *Hey2 *compound mutants in whole mount preparations and cross sections of the organ of Corti (Fig. 5). The precise rows of hair cells (Fig. 5A, C, D) are mirrored by corresponding parallel rows of supporting cells in the organ of Corti (Fig. 5B–D) [32,33]. As reported previously [34] an antibody against a homeodomain transcriptional factor, Prox1, distinctively labeled outer pillar cells (OP) between the inner and outer hair cells, and four rows of supporting cells that alternate with the three rows of outer hair cells (Fig. 5B–D). In *Hes5*^-/-^*;Hey2*^-/- ^samples, the ectopic row of outer hair cells (Fig. 5E, G, H) were accompanied by an additional row of supporting cells (Fig. 5F–H). In *Hes1*^-/-^*;Hey2*^+/- ^animals, the ectopic outer hair cells were also associated with additional supporting cells (Fig. 5I–N).

Since inner hair cell doublets were observed in *Hes1*^-/-^*;Hey2*^+/- ^animals, we also attempted to examine the arrangement of inner hair cells and its surrounding supporting cells in the mutant and wild-type cochleae (Fig. 5I–N). An antibody against p27/Kip1 marks all the supporting cells at this stage (Fig. 5J). However, the supporting cells surrounding inner hair cells labeled by p27/Kip1 at E18.5, the latest surviving stage for *Hes1 *and *Hey2 *compound mutants, showed variable arrangements in control cochleae (Fig. 5I, K). We could not determine whether the number of supporting cells surrounding inner hair cells changes accordingly in *Hes1 *and *Hey2 *compound mutants (Fig. 5L–N). Nevertheless, it is apparent that the ectopic inner hair cells in *Hes1*^-/-^*;Hey2*^+/- ^animals do not contact other inner hair cells and are interdigitated by non-hair cells as shown in whole mount preparations of the mutant organ of Corti (Fig. 3P–R), implying a preserved mosaic arrangement of inner hair cells and supporting cells in *Hes1*^-/-^*;Hey2*^+/- ^animals.

### No prolonged cell division was detected in the organ of Corti isolated from *Hey2 *mutants

**Figure 6:**
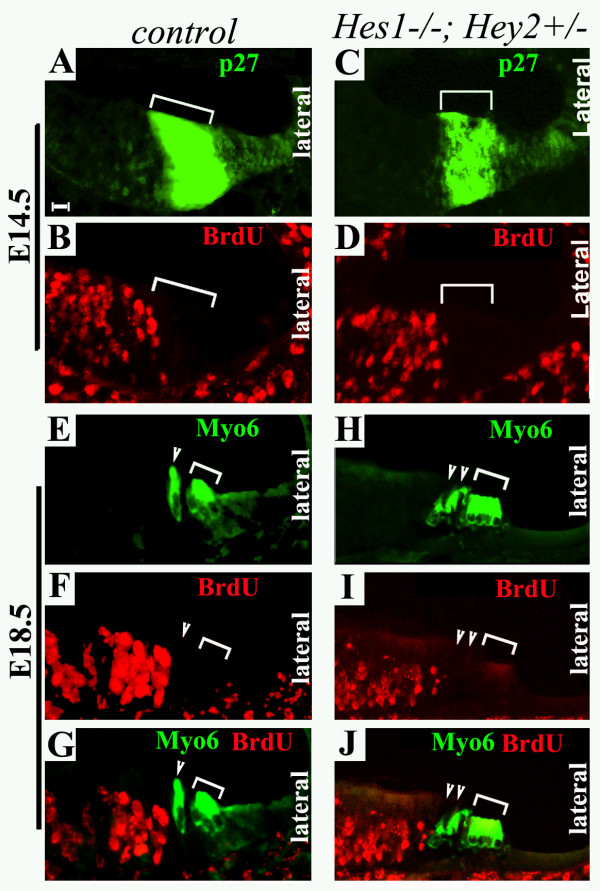
**The organ of Corti is postmitotic in *Hey2 *mutants by E14.5**. Adjacent cochlear sections from *Hey2*^+/- ^(**A**-**B, E-G**) and *Hes1*^-/-^*;Hey2*^+/- ^(**C**-**D, H-J**) at E14.5 (**A**-**D**) and E18.5 (**E**-**J**) were stained for antibodies against p27/Kip1 (**A**,**C**), BrdU (**B, D**), or doubly stained for Myosin6 and BrdU (**E**-**J**). Brackets in (**A**-**D**) indicate the primordial organ of Corti at E14.5 that is marked by the expression of p27/Kip1. Note that BrdU^+ ^proliferating cells are at the region medial to the developing organ of Corti at this stage. Brackets and arrowhead (**E**-**J) **indicate the outer and inner hair cells, respectively. Note that no BrdU^+ ^cells were detected in the organ of Corti following BrdU injections and a chasing period. Scale bar: 10 μm.

The mirror increase in supporting cells in *Hes5*^-/-^*;Hey2*^-/- ^and *Hes1*^-/-^*;Hey2*^+/- ^animals was not predicted from a simple lateral inhibition model [11], but consistent with an emerging complex role for the Notch signaling pathway. Indeed, the *Hes *homologous genes *Hes1 *and *Hes5 *can regulate sequential stages during neurogenesis in several nervous systems [31,35,36]. Initially, they define the domain undergoing neurogenesis and, subsequently, control the number of neural progenitors within the neurogenic domain. Notch signaling can affect cell division and/or cell fate determination to regulate the pool of progenitor cells at both stages [36-39]. In the cochlea, mutations in Notch receptor or ligands lead to perturbations in cell division [7,8]. Cell proliferation was observed in Notch or Notch ligand mutants after E14.5, when cells in the organ of Corti have withdrawn from the cell cycle in wild-type animals [8]. To determine whether prolonged cell division contributes to mis-patterning and ectopic hair cells and supporting cells in *Hey2*, *Hes1*, and *Hes5 *mutants, we analyzed the incorporation of a nucleotide analog, BrdU, in the organ of Corti from E14.5–E18.5 (Fig. 6, and data not shown).

BrdU was injected into timed-pregnant mice at E14.5, and embryos were collected the same day after three consecutive injections (Fig. 6A–D). In control mice, the organ of Corti was post-mitotic as demonstrated by the lack of BrdU incorporation in the region at E14.5 (Fig. 6A, B). No proliferating cells were detected in the primordial organ of Corti in *Hes1*^-/-^*;Hey2*^+/- ^embryos at E14.5 (Fig. 6C, D). Furthermore, to facilitate the identification of proliferating cells in the organ of Corti after E14.5, embryos injected with BrdU at E14.5 for three times were not collected until E18.5, allowing a longer chasing period for the incorporation of BrdU (Fig. 6E–J). Hair cells generated after E14.5 would be readily detected in embryos harvested at E18.5. The control E18.5 embryos had no BrdU^+ ^cells in the organ of Corti (Fig. 6E–G). In *Hes1*^-/-^*;Hey2*^+/- ^E18.5 embryos, no BrdU^+ ^cells were detected (Fig. 6H–J). Similar results were obtained with *Hes5 *and *Hey2 *compound mutants (data not shown), indicating that no detectable extension of cell proliferation contributes to the ectopic hair cells and supporting cells in *Hes1*^-/-^*;Hey2*^+/- ^(Fig. 6) or *Hes5*^-/-^*;Hey2*^-/- ^(data not shown) mutants. Since *Hes1*^-/-^*;Hes5*^-/- ^or *Hes1*^-/-^*;Hey2*^-/- ^embryos die early in development, we could not examine perturbation of cell division in these mutants.

## Discussion

Hes1 and Hes5 play essential roles in patterning inner and outer hair cells, respectively [14,15]. In addition, they appear to interact mutually for the patterning of both inner and outer hair cells in the development of the organ of Corti [15]. In this study, we show that the expression of Hey2 overlaps with that of *Hes1 *and *Hes5 *(Figs. 1 and 2) and that the patterning defects in the organ of Corti in *Hes1 *or *Hes5 *mutants were increased in *Hes1 *and *Hey2 *(Figs. 3 and 4), and *Hes5 *and *Hey2 *(Figs. 3 and 4) compound mutants. These data together suggested that all three genes interact genetically to regulate patterning of the organ of Corti.

Theoretically, the production of ectopic hair cells may be resulted from conversion of supporting cells to hair cells, prolonged cell proliferation, expansion of the boundaries of the developing postmitotic sensory organ, and/or an increased pool of progenitor cells. Previous studies indicate that prolonged cell proliferation occurs in Notch or Notch ligand mutants after E14.5 [8], in addition to possible conversion of some supporting cells to hair cells in these mutants [8]. It is not known whether an increased pool of progenitor cells, or expansion of the boundaries of the postmitotic sensory primordium may also contribute to the hyperplasia of the organ of Corti in these Notch or Notch ligand mutants. The expression of Hey2 in supporting cells during terminal differentiation (Fig. 2) is consistent with a role for mediating lateral inhibition, or repressing the cells surrounding hair cells to take on the same hair cell fate. Such a role would predict a loss of supporting cells to account for the ectopic hair cells in *Hey2 *mutants. Surprisingly, we not only did not see a decrease in supporting cells but also observed ectopic supporting cells associated with ectopic hair cells in *Hes1 *and *Hey2 *and in *Hes5 *and *Hey2 *compound mutants (Fig. 5). In addition, we did not detect any perturbation of cell division in *Hes1 *and *Hey2 *and in *Hes5 *and *Hey2 *compound mutants after E14.5 (Fig. 6 and data not shown).

These observations imply that an increased pool of progenitor cells prior to terminal mitosis and/or expansion of the boundaries of the postmitotic sensory primordium may account for the hyperplasia observed in *Hes1*;*Hey2 *and *Hes5*;*Hey2 *compound mutants. Consistent with either hypothesized roles, *Hey2 *also shows an early expression in the developing otocyst prior to the formation of the cochlea (Fig. 1B) and in the cochlea prior to the terminal differentiation of the organ of Corti (Figs. 1D, I), in addition to its expression in the supporting cells in the differentiating organ of Corti (Fig. 2C). At E11.5, the expression of *Hey2 *in the otocyst is excluded from the expression domain of Isl1 (Fig. 1A, B), which marks the neuronal and sensory lineages of the inner ear [24]. At E14.5 when hair cell differentiation has just begun in the inner hair cell region at the base of the cochlear duct, *Hey2 *is restricted to a domain that may be slightly medial to the primordial organ of Corti (Figs. 1D, I), and this expression of *Hey2 *in the cochlea is independent of Math1 (Fig. 1M). It is possible that, consistent with the dual role of Notch signaling in neurogenesis, Hey2 has a role in redundantly restricting the pools for neuroprogenitors or the regions for the sensori-neural lineages of the inner ear, and/or the boundaries of the sensory primordium. The small but quantitatively significant defect made it impossible to detect whether there is an enlarged progenitor pool, through extra cell divisions or expansion of the lineage boundaries prior to terminal mitosis, and/or whether the boundaries of the developing organ of Corti during terminal differentiation are expanded.

In comparison to recovered *Hes1*; *Hey2*, *Hes5*;*Hey2 *(Figs. 3, 4), or *Hes1*;*Hes5 *compound mutants [15], Notch or Notch ligand mutations cause a similar but much stronger patterning defect [7,8]. Early embryonic lethality associated with *Hes1*;*Hes5 *and *Hes1*;*Hey2 *compound mutants prevented us to recover double mutants carrying homozygous mutant alleles for *Hes1 *and *Hes5*, *Hes1 *and *Hey2*, or triple mutants. Hence, we were unable to conclusively ascertain whether *Hes1*, *Hes5 *and *Hey2 *are the key players mediating Notch signaling in the organ of Corti and whether additional players are involved. Indeed, an additional Hairy-related gene, *Hey1*, is also expressed in the organ of Corti during terminal differentiation (Fig. 1). It remains to be tested whether inactivation of other combinations or all of the *Hes *and *Hey *genes expressed in the developing cochlea by conditional inactivation will replicate the morphologic and molecular defects in Notch or Notch ligand mutants.

Cellular patterning defects also occur in planar cell polarity (PCP) mutants [33]. Cellular rearrangements characteristic of convergent extension occur during terminal differentiation of the organ of Corti and lead to extension of the cochlear duct [27,40,41]. In PCP signaling mutants, the process of convergent extension is inhibited. The cochlear extension and the organ of Corti patterning defects in PCP mutants are accompanied by misorientation of stereociliary bundles of hair cells [40,42]. The patterning defects in *Hes1*^-/-^*;Hey2*^+/- ^or *Hes5*^-/-^*;Hey2*^-/- ^mutants are distinctly different from that of PCP mutants. The extra row of outer hair cells or inner hair cell doublets in *Hes5*^-/-^*;Hey2*^-/- ^or *Hes1*^-/-^*;Hey2*^+/- ^mutants, respectively, were seen distributed along the entire length of the cochlear duct (Figs. 3, 4). No misorientation of stereociliary bundles and no shortening of the cochlear duct were observed in *Hes1*^-/-^*;Hey2*^+/- ^or *Hes5*^-/-^*;Hey2*^-/- ^mutants (data not shown). However, these cochlear duct defects have been observed in Notch and Notch ligand mutants that show significantly increased hair cell numbers in the unit length of the cochlear duct [7-9]. It is not clear whether inactivation of all the family members of Hey and Hes genes will replicate also the PCP-like cochlear duct defects of Notch mutants and whether the mis-patterning of hair cells in Notch and Notch ligand mutants is directly resulted from compromised convergent extension of the cochlear duct or a manifestation of altered cell fate determination and cell proliferation when Notch signaling is affected.

While members of the putative Notch target genes interact genetically in patterning the organ of Corti, their expressions show dynamically distinct, albeit overlapping, patterns in the developing cochlea. Accordingly, *Hey2 *and *Hes5 *appear to play a more important role in outer hair cells than in the inner hair cells while *Hey2 *and *Hes1 *act synergistically for correct patterning of the inner hair cells. These observations indicate a complex network of Notch target genes with temporal and spatially distinct and overlapping roles regulating the patterning of the organ of Corti and, possibly, cell lineage determination in the otocyst.

## Conclusion

In this study, we show that the expression of *Hey2 *overlaps with that of two Notch target genes, *Hes1 *and *Hes5 *(Figs. 1 and 2), and that the patterning defects in the organ of Corti in *Hes1 *or *Hes5 *mutants were increased in *Hes1*;*Hey2*, and *Hes5*; *Hey2 *compound mutants (Figs. 3 and 4). These data together suggested that *Hey2 *interacts genetically with *Hes1 *and *Hes5 *to regulate patterning of the organ of Corti. In addition, early embryonic lethality is observed in *Hey2*^-/-^;*Hes1*^-/- ^and *Hey2*^-/-^*;Hes1*^+/- ^mice, indicating that *Hey2 *interacts with *Hes1 *for early embryonic development and survival. We also explored the underlying molecular role for *Hey2 *and its family members, *Hes1 *and *Hes5*. We found that neither conversion of supporting cells (Fig. 5) to hair cells nor prolonged cell division (Fig. 6) can be detected to account for the hyperplasia of the organ of Corti in *Hey2 *compound mutants. It is possible that an increase in the progenitor pool, by accelerated cell proliferation and expansion of the boundaries of the sensory domain prior to terminal mitosis, and/or the expansion of the boundaries of the developing sensory organ during terminal differentiation, may result in production of ectopic hair cells associated with ectopic supporting cells.

## Methods

### Mouse strains and animal care

The animals used are *Hes1*^+/- ^(a gift from R. Kageyama) [31], *Hes5*^+/- ^(a gift from R. Kageyama) [31], *Hey2*^+/- ^[29], Math1/GFP [43], and *Math1*^+/- ^[5] mice. *Hes1*, *Hey2 *or *Hes5*, *Hey2 *compound mutant embryos were obtained from intercrosses of *Hes1*^+/-^*; Hey2*^+/- ^or *Hes5*^+/-^*; Hey2*^+/- ^doubly heterozygous mice. *Hey2*^+/- ^colonies were maintained in C57/B6 background. *Hes1*^+/- ^and *Hes5*^+/- ^mice in the CD1 background were backcrossed with C57/B6 mice for more than 8 generations for this study. The Hes1 and Hey2, and the Hes5 and Hey2 breeds are of a homogenous black coat color. *Math1*^-/- ^animals were generated by crossing *Math1*^+/- ^animals. Genotyping were carried out by PCR with tail DNA using the following oligonucleotides: Hes1, 5'-TGG GAT GEG GGA CAT GCG GG-3', 5'-TCA CCT CGT TCA TGA CTC G-3'; 5'-GCA GCG CAT CGC CTT CTA TC-3'; Hes5, 5'-GCT GGG GGC CGC TGG AAG TGG-3', 5'-CCG CTC CGC TCC GCT CGC TAA-3', 5'-GCA GCG CAT CGC CTT CTA TC-3'; and Hey2, 5'-CAC TAA GAA CTA GCG ATC TGG-3', 5' CTC AGG GGA TTT TGA AAG C-3', 5'GCA CGA GAC TAG TGA GAC GTG.

Animal care and use was in accordance with NIH guidelines and was approved by the Animal Care and Use Committee of Emory University and the University of California, Irvine. The morning after the mating of the animals is designated as embryonic day 0.5 (E0.5). The number of animals used for each genotype at each stage is least three and indicated where data are presented.

### BrdU injections

BrdU (Sigma) was dissolved at 5 mg/ml in 1× PBS and injected at 50 μg per gram of body weight. Mice were injected intraperitioneally three times at 2 hour intervals starting at E14.5 and sacrificed at the stages specified in the text.

### In situ hybridization

Inner ears were dissected from E11.5, E14.5, and E16.5 embryos and immediately fixed in 4 % paraformaldehyde (PFA) in PBS for 1 hour. Fixed inner ears were cryoprotected in 20% sucrose in PBS and embedded in OCT for cryostat sectioning. Sections (12–16 μm) were dried at room temperature for 1 hour and processed for in situ hybridization with digoxigenin-labeled probes [24]. Briefly, the inner ear sections were post-fixed in ice-cold 4% PFA for 10 minutes, treated with protease K (20 μg/ml) for 2–3 minutes, rinsed with DEPC-treated PBS, acetylated in 0.1 M TEA (Triethanolamine) freshly-made by adding 125 μl of acetic anhydride to 50 ml 0.1 M TEA, pH7.5, re-fixed in 4% PFA, and dehydrated by a series of EtOH (70%, 95%). The sections were then incubated with a hybridization solution (50% deionized Formamide, 10% Dextran Sulfate, 1× Denhardt's, 0.25 mg yeast tRNA, 0.3 M NaCl, 20 mM Tris-HCl, pH8.0, 5 mM EDTA, 10 mM NaPO4, pH7.2, 1% Sarcosyl) for 1 hour at 55° before incubated with the hybridization solution containing a probe (20 μg/ml) overnight at 55° in a sealed humidifier chamber. After the hybridization, slides were washed in pre-warmed washing solution (50% Formamide, 2×SSC) at 65° for three times, washed once with PBT (PBS, 0.1% Tween-20) at room temperature, blocked with 10% sheep serum in PBT for 1 hour at room temperature, incubated with alkaline phosphatase-conjugated anti-digoxigenin antibody in PBT, 1% sheep serum at 4° overnight. Following antibody incubation, slides were washed in NTMT (0.1 M NaCl, 0.1 M Tris-HCL, pH9.5, 0.05 M MgCl_2_, 0.1% Tween-20 containing Levamisole (0.5 mg/ml) for four times, and incubated with BM-purple AP substrate in NTMT without Levamisole for up to one week. Slides were washed in PBS and when appropriate color developed. Probes for *Hes1*, *Hes5*, *Hey1*, *Hey2*, *Oto90*, *Hes6*, and *Isl1 *were prepared using cDNAs cloned from the cochlea.

### Immunohistochemistry of cochlear sections and whole mount preparations

Immunostaining of cochlear sections were performed as described previously [24]. Primary antibodies used for this study are: anti-BrdU (Chemicon, mouse monoclonal, 1:100); anti-p27/Kip1 (BD Transduction Laboratories, mouse monoclonal, 1:200) anti-Myosin6 (Proteus BioSciences, rabbit polyclonal, 1:200); anti-Prox1 (Chemicon, rabbit polyclonal, 1:1000). Secondary antibodies are: FITC-conjugated donkey anti-rabbit (Jackson Lab, 1:1000); Rhodamine-conjugated donkey anti-goat (Jackson Lab, 1: 1000); Cy5-conjugated donkey anti-mouse (Jackson Lab, 1:1000). For p27/Kip1 and BrdU staining, sections were steamed in 10 mM NaCitrate for 15 minutes prior to being subjected to the staining procedure. For Prox1 staining, the samples were treated with 1% SDS followed by blocking and antibody incubations as described previously [24]. For hair cell staining of whole mount preparations of the cochlea, cochleae ducts were opened to expose the developing sensory epithelia prior to the staining procedure [40].

### Imaging, cell count and statistical analysis

Digital images were captured on an LSM 510 (Carl Zeiss, Germany) confocal laser scanning microscope and an inverted fluorescent microscope (Olympus, Japan). Images for entire cochlear ducts were composed using Photoshop (Adobe System, USA). The number of inner or outer hair cells in each composed cochlea was counted by scoring Myosin6^+ ^cells. The most distal tip of the apex region of the cochlea (10% of the length) was excluded from quantification since patterning of hair cells is incomplete in this region, and care was taken to use similarly 90% of the cochlear duct for each sample. Mis-patterned outer hair cells appearing as segments of an additional row of cells and inner hair cell doublets were counted. The number of mis-patterned inner and outer hair cells was directly plotted or the numbers of mis-patterned hair cells were calculated against normally patterned hair cells and plotted. Data collected from each experimental group with 3 samples from different animals are expressed as mean ± s.e.m. Student's t-test was used for statistical analysis.

## Abbreviations

IHC: inner hair cell; OHC: outer hair cell; E0.5: embryonic day 0.5; Delta-like1: Dll1; Jagged2: Jag2

## Authors' contributions

PC conceived and directed this study. SL contributed to most of the phenotypic analysis data, performed all the quantitative analyses, and wrote portions of the methods. SM carried out the initial phenotypic analyses, KRG and RS performed some ISH and IHC experiments. MTC provided inner ear samples and Hey2 null mice. The manuscript was written by PC, and edited and approved by all authors.
